# Protective Effects of Dexazoxane on Rat Ferroptosis in Doxorubicin-Induced Cardiomyopathy Through Regulating HMGB1

**DOI:** 10.3389/fcvm.2021.685434

**Published:** 2021-07-14

**Authors:** Haiyan Zhang, Zheng Wang, Zhengxia Liu, Kang Du, Xiang Lu

**Affiliations:** ^1^Department of Cardiology, The Second Affiliated Hospital, Nanjing Medical University, Nanjing, China; ^2^Department of Blood Transfusion, Sir Run Run Hospital, Nanjing Medical University, Nanjing, China; ^3^Department of Geriatrics, The Second Affiliated Hospital, Nanjing Medical University, Nanjing, China; ^4^Department of Geriatrics, Sir Run Run Hospital, Nanjing Medical University, Nanjing, China

**Keywords:** ferroptosis, dexazoxane, high mobility group box 3, heart failure, cardiology

## Abstract

Dexrazoxane (DXZ) reduces cytotoxicity caused by Doxorubicin (DOX). However, the mechanism of DXZ in ferroptosis and cardiomyopathy remains unclear. This research, therefore, explores the role and mechanism of DXZ in DOX-induced ferroptosis and cardiomyopathy in rats. Kaplan–Meier survival analysis was performed in rats treated by DOX in combination with ferroptosis inhibitor (FER-1) or other cell death–associated inhibitors. The ferroptosis, cardiotoxicity, and expression of high mobility group box 1 (HMGB1) in rats treated by DOX in combination with FER-1 or with DXZ were determined by hematoxylin and eosin staining, echocardiographic analysis, and quantitative real-time PCR. The ferroptosis in DOX-treated rats that received HMGB1 knockdown or overexpression was further detected using molecular experiments. Finally, the viability, level of malondialdehyde (MDA), and expressions of ferroptosis-related markers (PTGS2, GPX4, and FTH1) of rat cardiomyocyte H9c2 exposed to DOX combined with FER-1, zVAD (an apoptosis inhibitor), DXZ, or not were detected by performing molecular experiments. FER-1 increased the survival of the rats induced by DOX. The DOX-induced ferroptosis and cardiotoxicity could be reversed by FER-1 or DXZ. HMGB1 was induced by DOX but was inhibited by DXZ or FER-1. Overexpression of HMGB1 promoted the ferroptosis and cardiotoxicity induced by DOX in the rats although silencing of HMGB1 showed opposite effects. The data indicate that DOX suppressed the viability and increased the MDA level in H9c2 cells in a dose-dependent manner. Moreover, DOX-induced increase of PTGS2 and decrease of GPX4 and FTH1 in H9c2 cells was reversed by DXZ or FER-1. Therefore, DXZ has protective effects on ferroptosis and cardiomyopathy in rats through regulating HMGB1.

## Introduction

Doxorubicin (DOX) is an anticancer drug widely used in cancer treatments, including breast cancer (1), prostate cancer (2), and some other types of malignancies (3, 4). However, the clinical use of anthracyclines is limited due to their cardiotoxic effects, including irreversible degenerative cardiomyopathy and congestive heart (5). DOX induces injuries of multiple organs, for example, cardiomyopathy (6), which is a key pathogenic factor contributing to the development of lethal heart failure (7). Targeting topoisomerase 2 beta, mitochondrial impairment, and increasing the generation of reactive oxygen species (ROS) have been suggested as the underlying mechanisms of DOX-induced toxicity (8). Autophagic dysregulation has also been observed in DOX-induced cardiomyopathy (9, 10). The combination of DOX with other drugs, such as probucol, carvedilol, and antioxidant nutrients, has been previously shown to protect against DOX-induced cardiomyopathy (11). Nevertheless, a deeper understanding of the pathological processes in DOX-induced cardiotoxicity is still required.

Ferroptosis is a newly defined programmed cell death process characterized by the accumulation of iron-dependent lipid peroxides. Ferroptotic cell death also has unique morphological and bioenergetic features, including the shrinkage of the mitochondria, the increase of mitochondrial membrane density, the disruption of membrane integrity, and the depletion of intracellular NADH (12). A ferroptosis inducer in combination with chemotherapeutic DOX and actinomycin D could enhance the susceptibility in rhabdomyosarcoma and myoblast cells (13). However, whether the suppression of ferroptosis would produce protective effects on chemotherapeutic DOX-induced cardiomyopathy has been less reported. Recently, ferroptosis has been shown as a target for protecting against cardiomyopathy, where heme oxygenase-1 is involved in the progression (14). Meanwhile, it is also indicated in the study that apoptosis, necrosis, and autophagy inhibitors had no protective effect on the survival of mice with the induction of DOX (14), suggesting that ferroptosis may be the main factor causing DOX-induced death in mice. Nevertheless, the underlying mechanisms of ferroptosis in DOX-induced cardiomyopathy remain largely unclear. Thus, we were interested in investigating the role of ferroptosis in DOX-induced cardiomyopathy.

As an iron chelator, Dexrazoxane (DXZ) is currently the only drug approved by the Food and Drug Administration used on patients with cancers to prevent DOX-induced cardiotoxicity (15). It has also been revealed in previous research that DXZ can block the cytotoxicity caused by DOX in multiple diseases (16–18). Recently, it has been additionally demonstrated that the protective effects of DXZ on DOX-induced cardiotoxicity are involved in ferroptosis (14). However, the mechanism of DXZ in ferroptosis and DOX-induced rat cardiomyopathy remains largely unknown. High mobility group box 1 (HMGB1) is a ubiquitous and abundant nucleoprotein that plays a key role in many cardiovascular diseases, such as myocardial ischemia/reperfusion injury, myocardial infarction, and heart failure (19). HMGB1 was also found to be a critical regulator of ferroptosis in many diseases, such as leukemia (20), acute liver failure (21), and diabetic nephropathy (22). Therefore, we used a series of experiments *in vivo* and *in vitro* to explore whether the regulation of HMGB1 is implicated in the role of DXZ in DOX-induced cardiomyopathy.

In this study, DOX was used to induce the cardiomyopathy in rats, and it was found that DXZ had protective effects on DOX-induced cardiotoxicity, which was related to regulation of ferroptosis by targeting HMGB1.

## Materials and Methods

### Ethical Statement

The current research was approved by the Animal Research Ethics Committee of the Second Affiliated Hospital, Nanjing Medical University (No. H2016051930W).

### Animal and Drug Treatment

Male Wistar rats (250–300 g, *n* = 230) were purchased from Vitalriver. All the rats were kept in a specific pathogen-free room at 22–28°C under a 12-h light/dark cycle. Before the commencement of the experiments, the rats were fed with sterile food and water and acclimatized for 1 week.

Cardiomyopathy was induced in the rats using DOX according to previous research (14). Briefly, DOX (ST1285, Beyotime, China) was dissolved in sterile saline and intraperitoneally injected into the rats at a dose of 20 mg/kg, and the rats in the control group received an equiponderant saline injection only. One day before DOX injection, rats were intraperitoneally injected with ferroptosis inhibitor Ferrostain-1 (FER-1, 1 mg/kg, SML0583, Sigma, USA), necroptosis inhibitor Necrostain-1 (NEC-1, 1 mg/kg, 480,065, Sigma, USA), autophagy inhibitor 3-Methyladine (3-MA, 20 mg/kg, 189,490, Sigma, USA), or the pan-caspase inhibitor Emricasan (2.5 mg/kg, SML2227, Sigma, USA), respectively. Each inhibitor was dissolved in DMSO and diluted by sterile saline. To determine the role of HMGB1 in DOX-induced rat cardiomyopathy, a recombinant lentivirus expressing short hairpin RNA (shRNA) of HMGB1 (shHMGB1) and HMGB1 overexpression were purchased (Hanbio, China). One day before DOX injection, 5 × 10^7^ UT/50 μl lentivirus with shHMGB1 or overexpressed HMGB1 or the same volume of empty lentivirus (negative control, NC), was delivered into rats *via* tail vein injection. The target sequence for shHMGB1 was 5′-CGGAGGAAAATCAACTAAACATG-3′.

### Groups of Rats

Ten rats were recruited in each group in the experiment. The rats in the control, DOX, FER-1 + DOX, NEC-1 + DOX, 3-MA + DOX, and Emricasan + DOX groups were used for the overall survival analysis, and those from control and DOX groups were used for detection of the expression of PTGS2 in the indicated organs of rats. The rats from the control, DOX, DOX + FER-1, and DOX + DXZ groups were used to determine the effects of FER-1 and DXZ on DOX-induced cardiomyopathy, and those from the control, DOX, DOX + NC, DOX + shHMGB1, DOX + HMGB1, and DOX + HMGB1 + DXZ groups were used to explore the effects of HMGB1 on DOX-induced ferroptosis and cardiomyopathy. Furthermore, the rats from the control, DOX, DOX + DXZ, DOX + NC, DOX + HMGB1, and DOX + HMGB1 + DXZ groups were used to detect the effects of HMGB1 on the levels of heme and non-heme iron and the expression of iron-related genes.

### Hematological Parameters and MDA Content

Blood samples were collected 4 days after drug treatment, and the hematological parameters were measured using an ADVIA 2120i hematology analyzer (Siemens). The levels of malondialdehyde (MDA) in the serum and cardiac tissues of rats were detected 4 days after drug treatment using a commercial kit (S0131, Beyotime, China). The MDA levels in H9c2 cells were detected 24 h after drug treatment using the same kit.

### Quantitative Real-Time PCR

Total RNAs were isolated from the tissues or H9c2 cells using Trizol (15596018, Invitrogen, Thermofisher, USA), and the concentration and purity of RNA were measured using a spectrophotometer. The RNAs were reverse-transcribed using the PrimeScript™ II 1st Strand cDNA Synthesis Kit (6210B, Takara, Japan) in accordance with the instructions. Quantitative PCR was performed using a Bio-Rad CFX 96 Touch Real-Time PCR Detection System (1855196, Bio-Rad, China) and SYBR® Green PCR Master Mix (4312704, ABI, USA) following the instructions. Relative expressions of genes were calculated by the 2^−ΔΔCt^ method (23) and normalized to that of GAPDH. The parameters for quantitative real-time PCR (qRT-PCR) were set as follows: 95°C for 5 min, 40 cycles at 95°C for 15 s, at 60°C for 30 s, and at 70°C for 10 s. All reactions were conducted in triplicate. The primer sequences used are listed in Table 1.

**Table 1:** Sequences of primers used for real-time RT-PCR.

**Gene**	**Forward primer (5′-3′)**	**Reverse primer (5′-3′)**
GAPDH	CCGCATCTTCTTGTGCAGTG	GAGAAGGCAGCCCTGGTAAC
Ptgs2	ATGTTCGCATTCTTTGCCCAG	TACACCTCTCCACCGATGAC
Anp	CCCAGGCCATATTGGAGCA	CGCCCGAGAGCACCTC
Bnp	AGCTCTCAAAGGACCAAGGC	AAAACAACCTCAGCCCGTCA
Myh7	CAGTCATGGCGGATCGAGA	TGTCGAACTTGGGAGGGTTC
HMGB1	CACCCTGCATATTGTGGTAGG	CGCTGGGACTAAGGTCAACA
Tfrc	AGGTGCTTCAGAGTGCTCCC	AGCCAGTCTCACACACTCCT
Fth1	CCCTTTGCAACTTCGTCGCT	CTCCGAGTCCTGGTGGTAGT

### Hematoxylin and Eosin Staining

The hearts of rats were fixed in 4% paraformaldehyde overnight, then embedded in paraffin, and finally sectioned into 5-μm-thick slices. The sections were dewaxed, hydrated, and stained by hematoxylin at room temperature for 5 min, followed by eosin staining at room temperature for 30 s. Finally, the zones with the pathological lesions were detected under a light microscope (TS100, Nikon, Japan).

### Echocardiography

A Visual Sonics Vevo770 Imaging System was used to analyze the transthoracic echocardiography as previously described (24). The left ventricular ejection fraction (LVEF) and left ventricular fractional shortening (LVFS) of rats in each group were recorded and evaluated.

### Immunohistochemistry

The sections were deparaffinized, blocked by phosphate-buffered saline (PBS) containing 5% (v/v) normal goal serum and 1% (w/v) BSA, and then incubated at 4°C overnight with the antibodies against HMGB1 (1:200, ab79823, Abcam, USA) or 4-HNE (ab46545, 1:200; Abcam, USA) under humidified conditions, and the sections were then further incubated with antirabbit secondary antibody (diluted at 1:500, Proteintech) at room temperature for 1 h. The images of immunohistochemistry were captured using Eclipse E400 microscope (Nikon, Japan), and the stained areas were analyzed by ImageJ 1.48u (National Institutes of Health, USA).

### Heme and Non-heme Iron Measurement

The levels of heme in the serum and tissue were measured using the Heme Assay Kit (MAK316, Sigma, USA) following the instructions. In brief, heme in 50 μl of serum or tissue homogenate was added to a 96-well plate. Then, 200 μl of the reagent for the detection of heme was added into each well, and the absorption at 400 nm was recorded using a SpectraMax Plus 384 Microplate Reader (PLUS 384, Molecular Devices, USA). The level of heme was calculated according to the standard curve of known heme concentrations.

The chromogen method was used to detect the level of non-heme iron in the tissue (25). Tissues were weighed and digested in NHI acid (10% trichloroacetic acid in 3 M HCl) at 65–70°C for 48 h. Equal volumes of sample or standard iron (500 μg/dl) were incubated at room temperature for 10 min in 200 μl BAT buffer (0.2% thioglycolic acid and 0.02% disodium-4,7-diphenyl-1,10-phenanthroline disulfonate in 50% saturated NaAc solution). The absorption at 535 nm was read using a microplate reader, and the level of non-heme iron was calculated according to a standard curve from the iron standard. The data are shown as micrograms of iron per gram of wet tissue weight. For the isolation of serum, the blood samples were diluted to 1:10, collected and transferred onto ice, and then centrifuged at 1,800 × g, 4°C for 10 min. Iron was extracted from the serum before the use of ethylenediaminetetraacetic acid and trichloroacetic acid as described in a prior study (26). The spectra of atomic absorption were acquired from a Varian SpectrAA 220Z graphite furnace atomic absorption spectrometer. The level of serum non-heme iron was calculated according to a standard curve from the iron standard.

### Cell Culture and *In vitro* Treatment

H9c2 rat cardiomyocytes (CBP60588, Cobioer, China) were cultured in Dulbecco's modified Eagle's medium (11966025, Gibco, ThermoFisher, USA) supplemented with 10% heat-inactivated fetal bovine serum (16140071, Gibco, ThermoFisher, USA) and 1 × penicillin-streptomycin (10378016, Gibco, ThermoFisher, USA). The cells were incubated at 37°C in a humidified atmosphere containing 5% CO_2_ and 95% air. The cells were treated by DOX at different concentrations (0.5, 1, 2, and 4 μM) and by inhibitors [FER-1 (10 μM), DXZ (100 μM), and caspase inhibitor zVAD (20 μM, V116, Sigma, USA)].

### MTT Assay

H9c2 cells (5 × 10^3^ cells per well) were treated with or without DOX in the presence or absence of zVAD, DOX, or FER-1 for 72 h, respectively, and then seeded in a 96-well plate. After culture for 48 h, 10 μl MTT reagent (C0009, Beyotime, China) was added into each well. The cells were incubated with MTT reagent at 37°C for 4 h. Then, 100 μl Formazan solving reagent was added into each well to dissolve the formazan precipitation. In the end, the absorbance of each well at the wavelength of 570 nm was read by the SpectraMax Plus 384 Microplate Reader (PLUS 384, Molecular Devices, USA).

### Western Blot

Total proteins were extracted from the tissues or cells using RIPA buffer (89901, Thermo Scientific, USA) containing protease inhibitors (36978, Thermo Scientific, USA). The supernatant was then collected by centrifugation at 4°C for 30 min at 12,000 × g. The concentration of the protein was measured using the BCA Protein Assay Kit (P0012S, Beyotime). The proteins (50 μg per sample) were isolated on 10–12% SDS-PAGE and then transferred to a polyvinylidene difluoride membrane (LC2002, Invitrogen, ThermoFisher, USA), which was blocked by 5% non-fat milk (PA201-01, BioMed, China) at room temperature for 1 h. The following primary antibodies were used for subsequent incubation: anti-PTGS2 antibody (ab15191, Abcam, UK); anti-HMGB1 antibody (ab18256, Abcam, UK); anti-GPX4 antibody (ab125066, Abcam, UK); anti-FTH1 antibody (ab183781, Abcam, UK) and anti-GAPDH antibody (ab8245, Abcam, UK). After incubation with the primary antibodies at 4°C overnight, the membrane was washed and probed by appropriate HRP-conjugated antirabbit IgG antibody (1:5,000, 7,074, Cell Signaling Technology, USA) for PTGS2, GPX4, and FTH1 or HRP-conjugated antiMouse IgG antibody (1:5,000, 70-GAM007, MultiSciences, China) at room temperature for 2 h. GAPDH served as an internal control. SignalFire™ ECL reagent (6,883, Cell Signaling Technology, USA) was used to detect the signals.

### Lipid Peroxidation Assay

The lipid peroxidation of cell lysates was detected by measuring the formation rate of TBAR and expressed as pmol/mg protein. After culture for 48 h, H9c2 cells were washed twice with PBS and lysed with lysis buffer. After the centrifugation, the supernatant was collected for MDA assay according to the manufacturer's instructions. The absorbance was measured by a microplate reader (SpectraMax, CA, USA). The concentrations of protein were determined by a BCA protein assay kit.

### Intracellular Ferrous Ion Determination

Cells were collected to measure the intracellular Fe^2+^ by commercial assay kits (27), which were purchased from Abcam (ab83366, Cambridge, MA) according to the instructions of the manufacturer.

### Lactate Dehydrogenase Release Assay

Lactate dehydrogenase (LDH) leakage was conducted to determine cell injury. The released LDH was detected in the collected culture medium by spectrophotometry by the CytoTox 96 non-Radioactive Cytotoxicity Assay kit (Promega, Madison, USA).

### Apoptosis Assay

H9c2 cells (5 × 10^3^ cells per well) were treated with or without DOX in the presence or absence of DOX or FER-1 for 72 h. After culture for 48 h, flow cytometry was used to detect the apoptosis in the cells. In short, the cells were washed twice with PBS and centrifuged to remove the supernatant. About 400 μl buffer was added to make the cell suspension. Annexin V (5 μl) and propidium iodide (5 μl) buffer were added to the cell suspension. Following the incubation in darkness at 37°C for 20 min, apoptosis of the cell was detected by flow cytometry with the help of a flow cytometer (BD, San Jose, CA).

### Statistical Analysis

The data were analyzed and graphed using GraphPad Prism 5.02 software (La Jolla, CA, USA) and are shown as mean ± SD. The Shapiro–Wilk-test was used to detect the normal distribution. Student's *t*-test or one-way ANOVA followed by Tukey's *post-hoc* test was used for statistical analysis as appropriate. For the Kaplan–Meier survival plots, statistical significance was measured by log-rank (Mantel–Cox) test. A *P*-value < 0.05 was considered to be a significant difference. All experiments were repeated independently three times.

## Results

### FER-1 Was Found to Decide DOX-Induced Mortality

**Figure 1:**
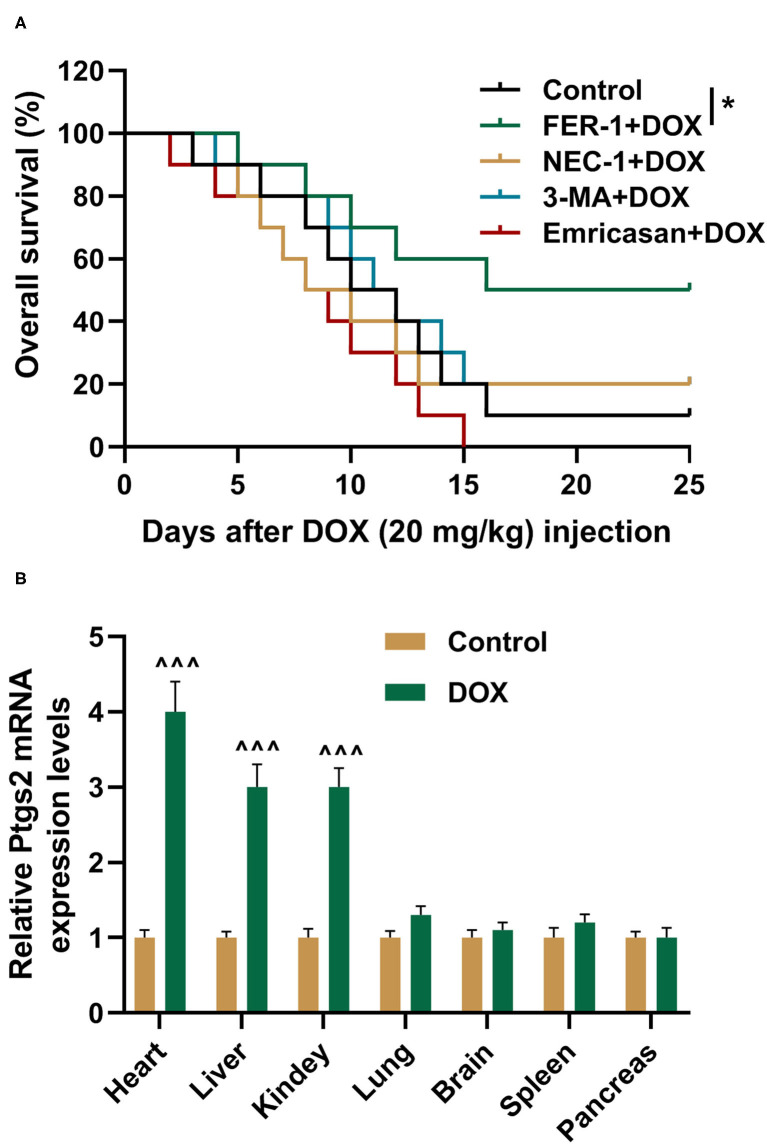
FER-1 is found to decide DOX-induced mortality. **(A)** The survival rate of rats from different groups was shown by Kaplan–Meier survival analysis. All rats were injected with DOX (20 mg/kg). Ten rats were recruited in each group. **(B)** qRT-PCR analysis shows the mRNA expression level of PTGS2 in the indicated organs of rats after injection with saline (Control) and DOX (20 mg/kg) for 4 days with 10 rats in each group. Each experiment was repeated three times. ^∧∧∧^*P* < 0.001 vs. control. *P < 0.05 vs. DOX. DOX, doxorubicin; FER-1, a ferroptosis inhibitor; NEC, a necrosis inhibitor; 3-MA, an autophagy inhibitor; Emricasan, a pan-caspase inhibitor.

To verify the roles of ferroptosis, apoptosis, and autophagy in DOX-induced mortality, the rats were pretreated by saline, FER-1, NEC-1, 3-MA, or Emricasan. Then, the rats were treated by DOX at 20 mg/kg, and their survival times were detected using Kaplan–Meier survival plots. It was indicated in the result that FER-1 treatment could increase the survival times of the rats treated by DOX (Figure 1A, *P* < 0.05). Meanwhile, treatment with NEC-1, 3-MA, or Emricasan had no positive effects on the survival times of DOX-treated rats (Figure 1A). Prostaglandin-endoperoxide synthase 2 (PTGS2) is a molecular marker of ferroptosis (28), and its expression was detected by qRT-PCR in the indicated organs of rats. We found that DOX induced the increased expression of PTGS2 in the heart, liver, and kidney of rats although it had no effect on the expression of PTGS2 in other indicated organs (Figure 1B).

### Protective Effects of FER-1 and DXZ on DOX-Induced Cardiomyopathy

**Figure 2:**
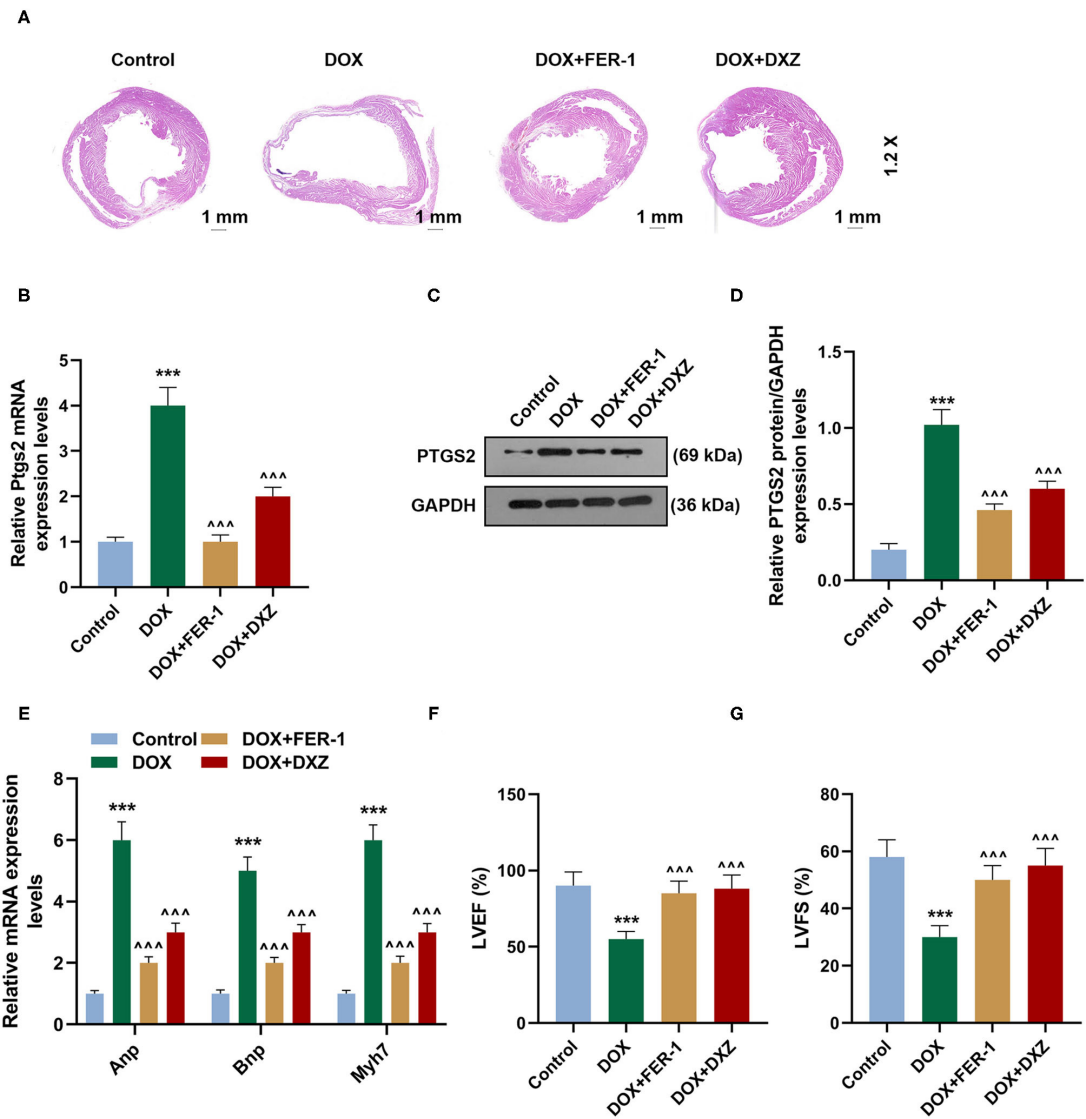
Protective effects of FER-1 and DXZ on DOX-induced cardiomyopathy. **(A)** Representative images of HE staining of heart sections in rats from different groups. Magnification: ×1.2. Scale bar = 1 mm with 10 rats in each group. **(B–D)** qRT-PCR and Western blot analysis show the mRNA and protein level of PTGS2, in heart tissues of rats with 10 rats in each group. Three mechanical duplications were performed in each sample. **(E)** qRT-PCR analysis show the mRNA expression of ANP, BNP and MYH7 in heart tissues of rats. **(F,G)** Echocardiographic analyses of cardiac function in control rats and rats treated by DOX with or without FER-1 or DXZ. Ten rats were included in each group. ***P < 0.001 vs. control. ^∧∧∧^*P* < 0.001 vs. DOX. HE, hematoxylin-eosin; DXZ, dexazoxane; PTGS2, prostaglandin-endoperoxide synthase 2; ANP, natriuretic peptide A; BNP, natriuretic peptide B; MYH7, myosin heavy chain 7; qRT-PCR, quantitative real-time PCR; LVEF, left ventricular ejection fraction; LVFS, left ventricular fractional shortening.

Furthermore, to investigate the effect of DXZ on DOX-induced ferroptosis in rats, rats were treated with saline (control), DOX, or DOX combined with FER-1 or DXZ. It was indicated in the results of H&E staining that DOX treatment induced cardiomyopathy, and such effect on the heart was alleviated by treatment with DXZ or FER-1 (Figure 2A). The expression of PTGS2 mRNA and protein detected by qRT-PCR and Western blot in the heart tissues of rats treated by DOX were significantly increased although FER-1 and DXZ could obviously inhibit the DOX-induced increase on the expression of PTGS2 mRNA and protein (Figures 2B–D, *P* < 0.001). As ANP, BNP, and MYH7 are all classic biomarkers of cardiac hypertrophy (29, 30), we also detected their expressions in heart tissues of rats from different groups. The qRT-PCR results indicate that the expressions of ANP, BNP, and MYH7 were all upregulated by DOX, and the DOX-induced increase on the expressions of ANP, BNP, and MYH7 could be reversed by FER-1 and DXZ (Figure 2E, *P* < 0.001). Next, we also evaluated the cardiac function in rats of different groups using echocardiographic analyses and found that both LVEF and LVFS were reduced in the DOX-treated rats, and such decreases induced by DOX could be reversed by treatment using FER-1 and DXZ (Figures 2F,G, *P* < 0.001).

### HMGB1 Was Essential for DOX-Induced Ferroptosis and Cardiotoxicity

**Figure 3:**
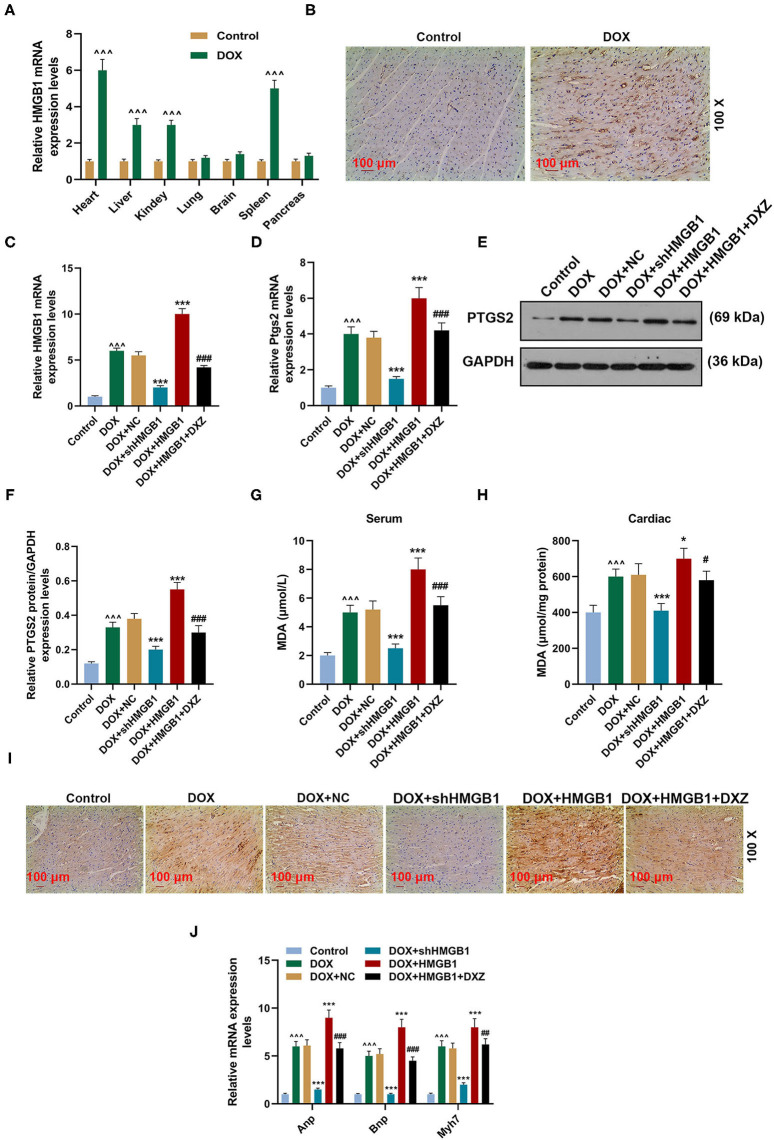
HMGB1 is essential for DOX-induced ferroptosis and cardiotoxicity. **(A)** qRT-PCR analysis shows the mRNA expression level of HMGB1 in the indicated organs of rats after injection with saline (control) and DOX (20 mg/kg) with 10 rats in each group. **(B)** The expression of HMGB1 in heart tissues of rats from the control and DOX groups was detected by immunohistochemistry. Magnification: ×100. Scale bar = 100 μm. Ten rats were used in each group. **(C–F)** qRT-PCR and WB analysis show the expression level of PTGS2 mRNA and protein and HMGB1 mRNA in rat heart tissues. Ten rats were used in each group. **(G,H)** The level of MDA in the serum and cardiac tissue of rats was measured by a lipid peroxidation MDA assay kit. Ten rats were used in each group. **(I)** The expression of 4-HNE in the heart tissues of rats from the control, DOX, DOX+NC, DOX+shHMGB1, DOX+HMGB1 and DOX+HMGB1+DXZ groups was detected by immunohistochemistry. Magnification: ×100. Scale bar = 100 μm. Ten rats were used in each group. **(J)** The expressions of ANP, BNP, and MYH7 in heart tissues of rats were calculated by qRT-PCR. Ten rats were used in each group. ^∧∧∧^*P* < 0.001 vs. control. *P < 0.05, ***P < 0.001 vs. DOX. ^#^*P* < 0.05, ^##^*P* < 0.01, ^###^*P* < 0.001 vs. DOX + HMGB1. Each experiment was repeated three times. HMGB1: high mobility group box 1; MDA: malondialdehyde; 4-HNE: 4-hydroxynonenal.

To explore the role of HMGB1 in DOX-induced ferroptosis and cardiotoxicity, qRT-PCR and immunohistochemistry were performed to determine the expression of HMGB1 in rats. The qRT-PCR results indicate that the expression of HMGB1 was significantly upregulated in the heart, liver, kidney, and spleen tissues of the rats treated by DOX (Figure 3A, *P* < 0.001), and there was no change in the expression of HMGB1 in the lung, brain, and pancreas tissues (Figure 3A). Immunohistochemistry analysis of HMGB1 in rat heart tissues also suggested that DOX obviously induced the expression of HMGB1 within the heart tissue (Figure 3B).

The expression of HMGB1 and PTGS2 was detected again to further investigate the effect of HMGB1 and DXZ on ferroptosis, and it was indicated in the qRT-PCR and Western blot results that DOX induced the increase on the expression of HMGB1 and PTGS2 in heart tissues, and knocking down HMGB1 could attenuate such an increase on the expression of HMGB1 and PTGS2 induced by DOX (Figures 3C–F, *P* < 0.001). The overexpression of HMGB1 combined with DOX treatment promoted the upregulation of HMGB1 and PTGS2, which could be partly blocked by DXZ (Figures 3C–F, *P* < 0.001). Next, the level of MDA, the lipid peroxidation-associated byproduct, in the serum or cardiac tissue of rats was detected, and the result indicates that DOX caused the increase of MDA in both serum and cardiac tissue of rats (Figures 3G,H, *P* < 0.001). Polyunsaturated fatty acids in mitochondrial membranes are the primary targets for ROS attack and could lead to lipid peroxidation and the generation of reactive lipids, such as 4-hydroxynonenal (4-HNE) (31). Thus, the level of 4-HNE was detected to assess the degree of lipid peroxidation in the cardiac tissue of rats, and we found that DOX treatment increased the level of 4-HNE and that silencing HMGB1 could significantly inhibit the expression of 4-HNE. Moreover, the overexpression of HMGB1 combined with DOX treatment increased the level of 4-HNE, which could be partly weakened by DXZ (Figure 3I).

**Figure 4:**
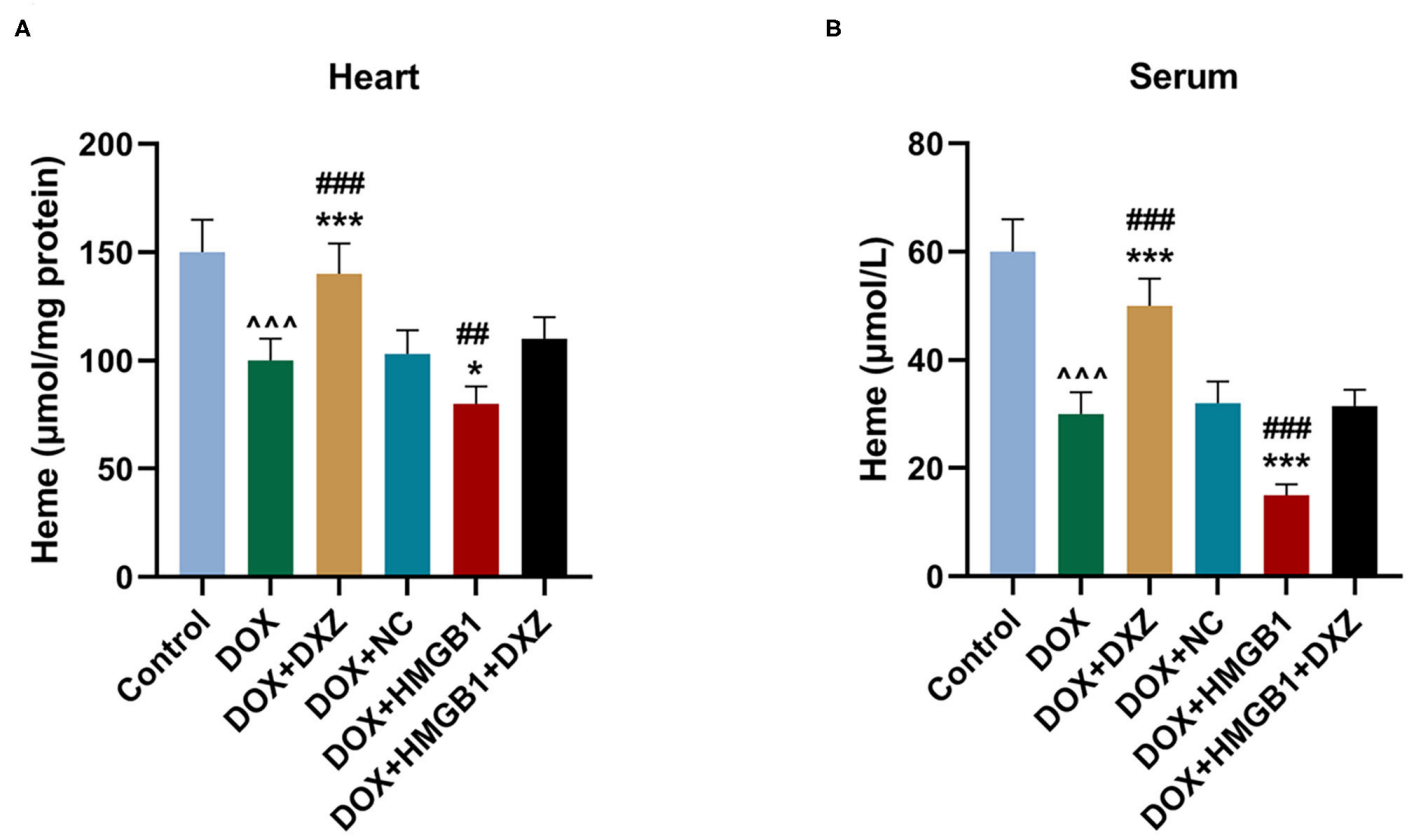
The levels of heme in the heart and serum of rats. **(A,B)** The level of heme in the heart and serum of rats from different groups after the treatment for 4 days was detected by a commercial kit. Ten rats were used in each group. ^∧∧∧^*P* < 0.001 vs. control. *P < 0.05, ***P < 0.001 vs. DOX. ^##^*P* < 0.01, ^###^*P* < 0.001 vs. DOX + HMGB1 + DXZ. Each experiment was repeated three times.

**Figure 5:**
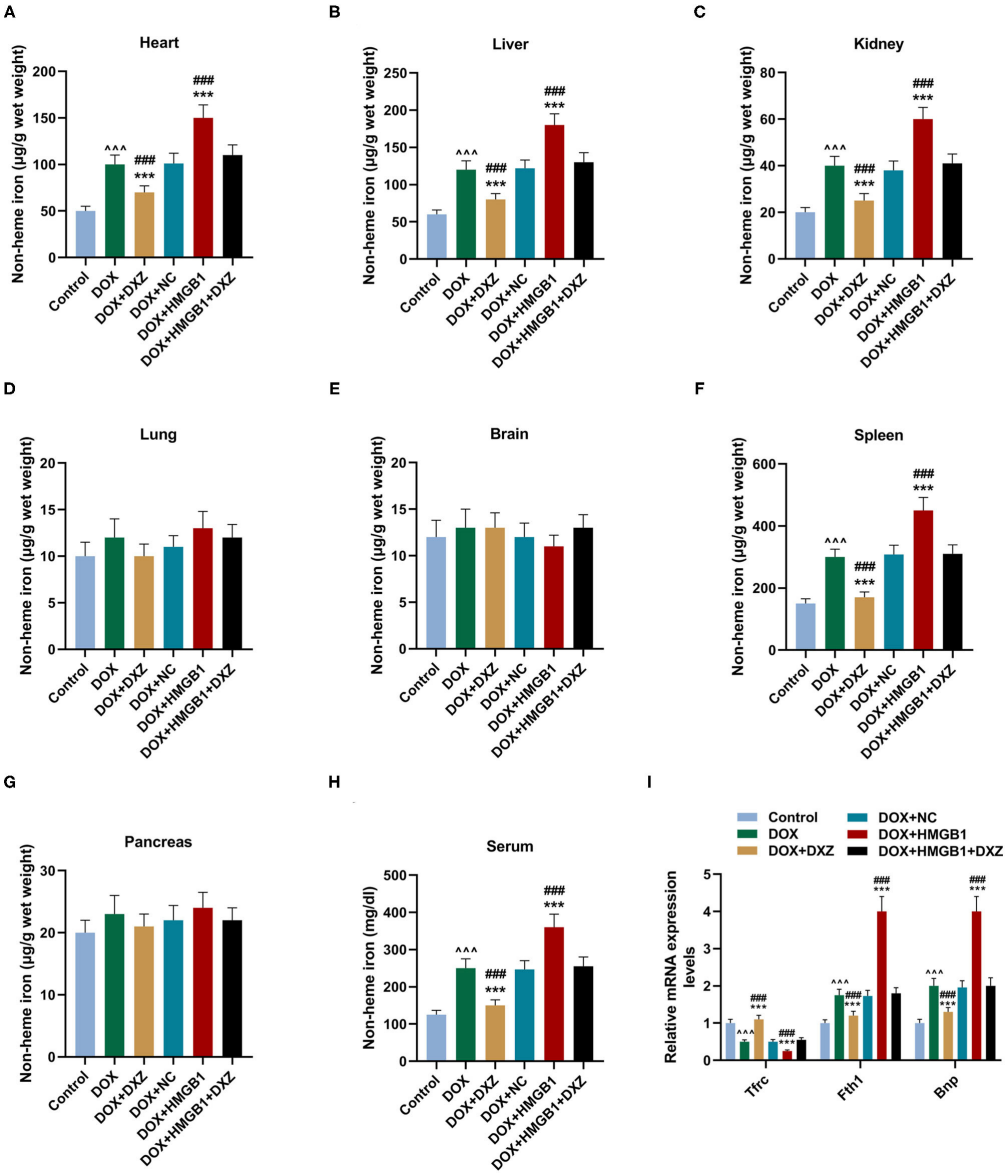
The levels of non-heme iron in the indicated organs and serum of rats. **(A–H)** The levels of non-heme iron in the indicated organs and serum of rats from different groups at 4 days after the treatment was detected by a commercial kit. Ten rats were used in each group. **(I)** The cardiac mRNA expression levels of TFRC, FTH1, and BNP were quantified by qRT-PCR. Ten rats were used in each group. ^∧∧∧^*P* < 0.001 vs. control. ***P < 0.001 vs. DOX. ^###^*P* < 0.001 vs. DOX + HMGB1 + DXZ. Each experiment was repeated three times.

Furthermore, the biomarkers of cardiac hypertrophy, the expressions of ANP, BNP, and MYH7 in rats from different groups, were detected by qRT-PCR, and according to the data, it is indicated that DOX induced the increase in the expression of the biomarkers of cardiac hypertrophy, which was enhanced by the overexpression of HMGB1 and inhibited by the knockdown of HMGB1 and treatment using DXZ (Figure 3J, *P* < 0.01). As heme degradation is the principal mechanism underlying the effects of DOX treatment (14), therefore, the levels of heme and non-heme iron were detected in heart and serum of rats from the control, DOX, DOX + DXZ, DOX + NC, DOX + HMGB1, and DOX + HMGB1 + DXZ groups (Figures 4, 5). DOX reduced heme level in heart and serum of rats (Figures 4A,B, *P* < 0.001); however, the level of non-heme iron in the heart, liver, kidney, spleen, and serum was increased by DOX (Figures 5A–H, *P* < 0.001). However, overexpression of HMGB1 enhanced the effects of DOX on the levels of heme and non-heme iron in rats, and DXZ could diminish the effects of DOX on the levels of heme (Figures 4A,B, *P* < 0.001) and non-heme iron (Figures 5A–H, *P* < 0.001). We further measured the hematological parameters in control- and DOX-treated rats, and it is illustrated in the analysis of hematologic parameters that the number of white blood cells and hemoglobin was decreased, yet the platelet count was increased by DOX (Table 2). Furthermore, the expressions of iron-related genes (TFRC, FTH1, and BNP) in the hearts of rats were determined, and TFRC expression was found to be downregulated by DOX in rat heart tissues, and those of FTH1 and BNP were upregulated by DOX (Figure 5I, *P* < 0.001). Meanwhile, it was also found that DXZ suppressed the effect of DOX on the expressions of TFRC, FTH1, and BNP although HMGB1 could enhance the effect of DOX on the expressions of TFRC, FTH1, and BNP in heart tissues of rats (Figure 5I, *P* < 0.001).

**Table 2:** Hematologic parameters in rats treated with DOX or not.

	**Blank (*n* = 10)**	**DOX (20 mg/kg) (*n* = 10)**	***P***
WBC (10^9^/L)	2.5 ± 0.58	1.25 ± 0.35	<0.001
RBC (10^12^/L)	9.48 ± 0.62	9.84 ± 1.02	0.35
HGB (g/dL)	16.22 ± 0.78	15.31 ± 0.54	<0.01
HCT (%)	48.24 ± 4.82	52.89 ± 6.98	0.10
MCV (fL)	52.24 ± 6.62	55.88 ± 4.62	0.10
MCH (Pg)	16.68 ± 0.72	17.02 ± 0.64	0.28
MCHC (g/dL)	32.28 ± 4.41	30.13 ± 2.23	0.19
PLT (10^9^/L)	1205.36 ± 148.25	1598.86 ± 162.38	<0.001

*Data was displayed as mean ± SEM. WBC, white blood cell count; RBC, red blood cell count; HGB, hemoglobin; HCT, hematocrit; MCV, mean corpuscular volume; MCH, mean corpuscular hemoglobin; MCHC, mean corpuscular hemoglobin concentration; PLT, platelet count*.

### Effects of HMGB1 and DXZ on DOX-Treated Injury of H9c2 Rat Cardiomyocytes

**Figure 6:**
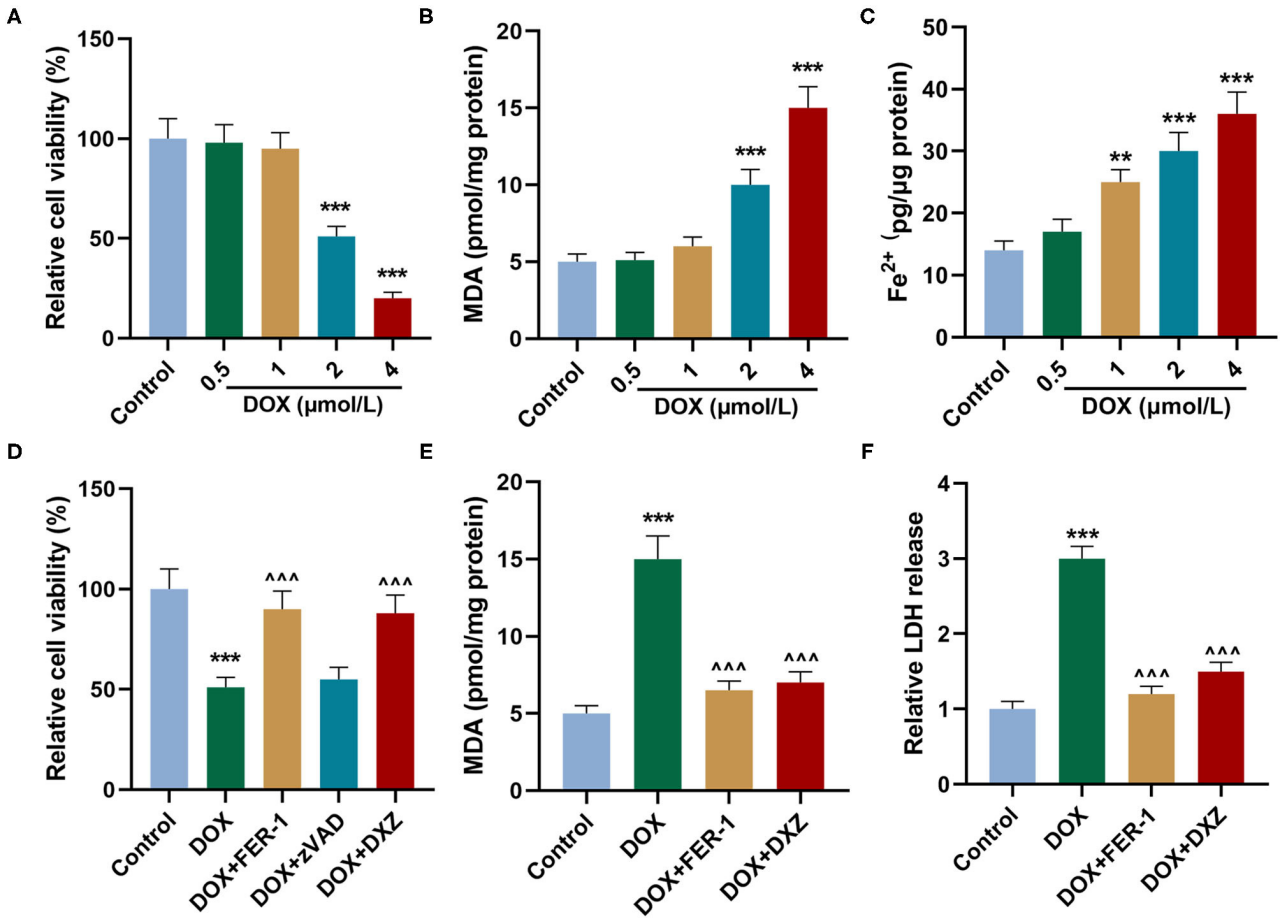
Effects of HMGB1 and DXZ on DOX-treated injury of H9c2 rat cardiomyocytes. **(A)** The viability of H9c2 cells after the incubation of DOX for 12 h at 0 (control), 0.5, 1, 2, and 4 μmol/L was detected by MTT assay. **(B)** The levels of MDA in H9c2 cells after the incubation of DOX for 12 h at 0 (control), 0.5, 1, 2, and 4 μmol/L were detected by a commercial kit. **(C)** The Fe^2+^ content of H9c2 cells after the incubation of DOX for 12 h at 0 (control), 0.5, 1, 2, and 4 μmol/L was detected by assay kits. **(D)** The viability of H9c2 cells after drug treatment in the control group or DOX-treated cells after incubation of FER-1 (10 μmol/L), zVAD (20 μmol/L, an apoptosis inhibitor), or DXZ (100 μmol/L) for 72 h was detected by MTT assay. **(E)** The MDA levels in H9c2 cells after drug treatment for 24 h were detected by a commercial kit. **(F)** The LDH levels in H9c2 cells after drug treatment for 24 h were detected by a commercial kit. **P < 0.01, ***P < 0.001 vs. control. ^∧∧∧^*P* < 0.001 vs. DOX. Each experiment was repeated three times.

**Figure 7:**
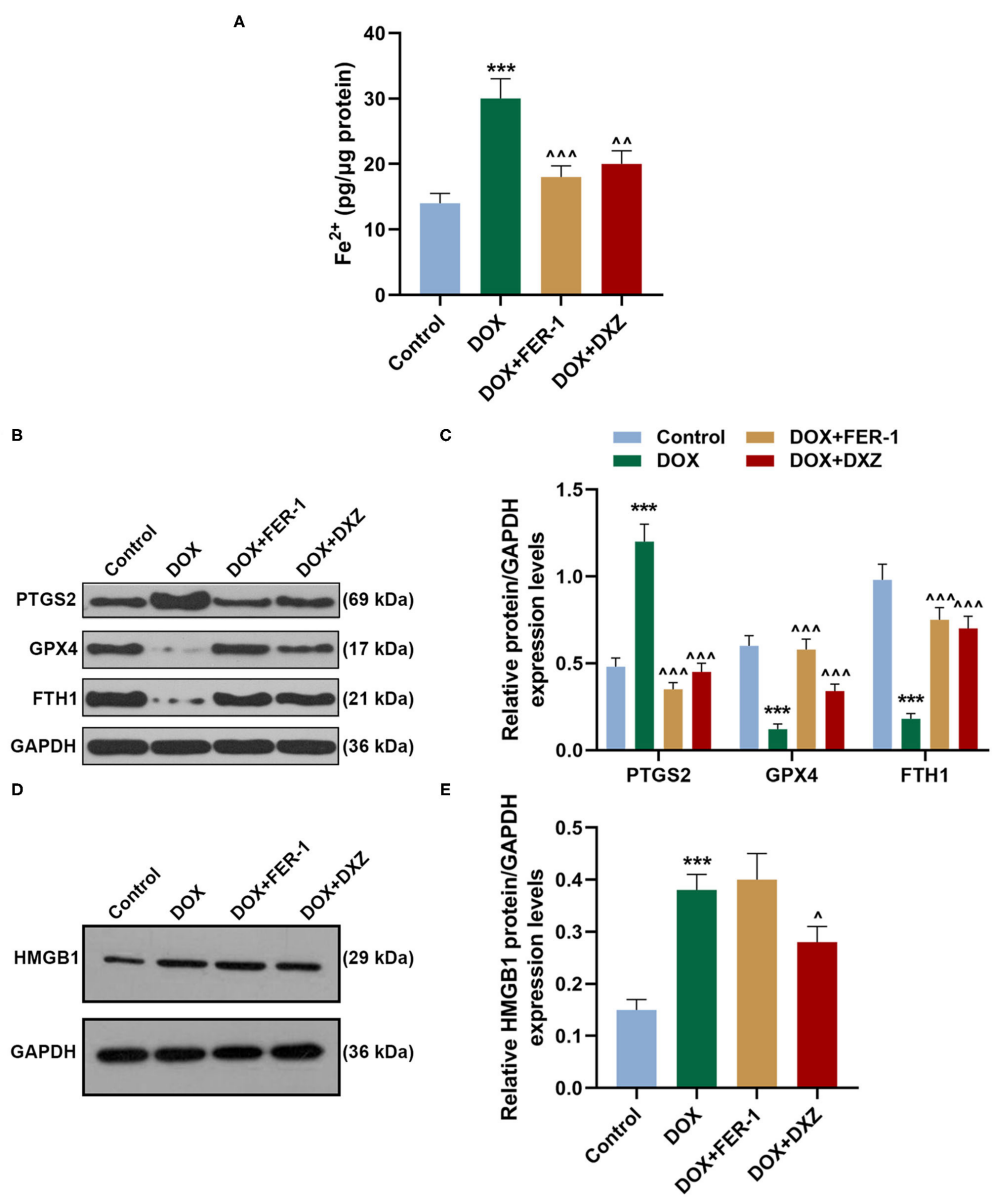
Effects of HMGB1 and DXZ on DOX-induced ferroptosis of H9c2 rat cardiomyocytes. **(A)** The Fe^2+^ content of H9c2 cells after drug treatment was detected by assay kits. **(B–E)** The relative protein expression levels of HMGB1, PTGS2, GPX4, and FTH1 were determined by Western blot. GAPDH served as a reference gene. ***P < 0.001 vs. control. ^∧^*P* < 0.05, ^∧∧^*P* < 0.01, ^∧∧∧^*P* < 0.001 vs. DOX. All experiments were repeated three times. GPX4, glutathione peroxidase-4.

**Figure 8:**
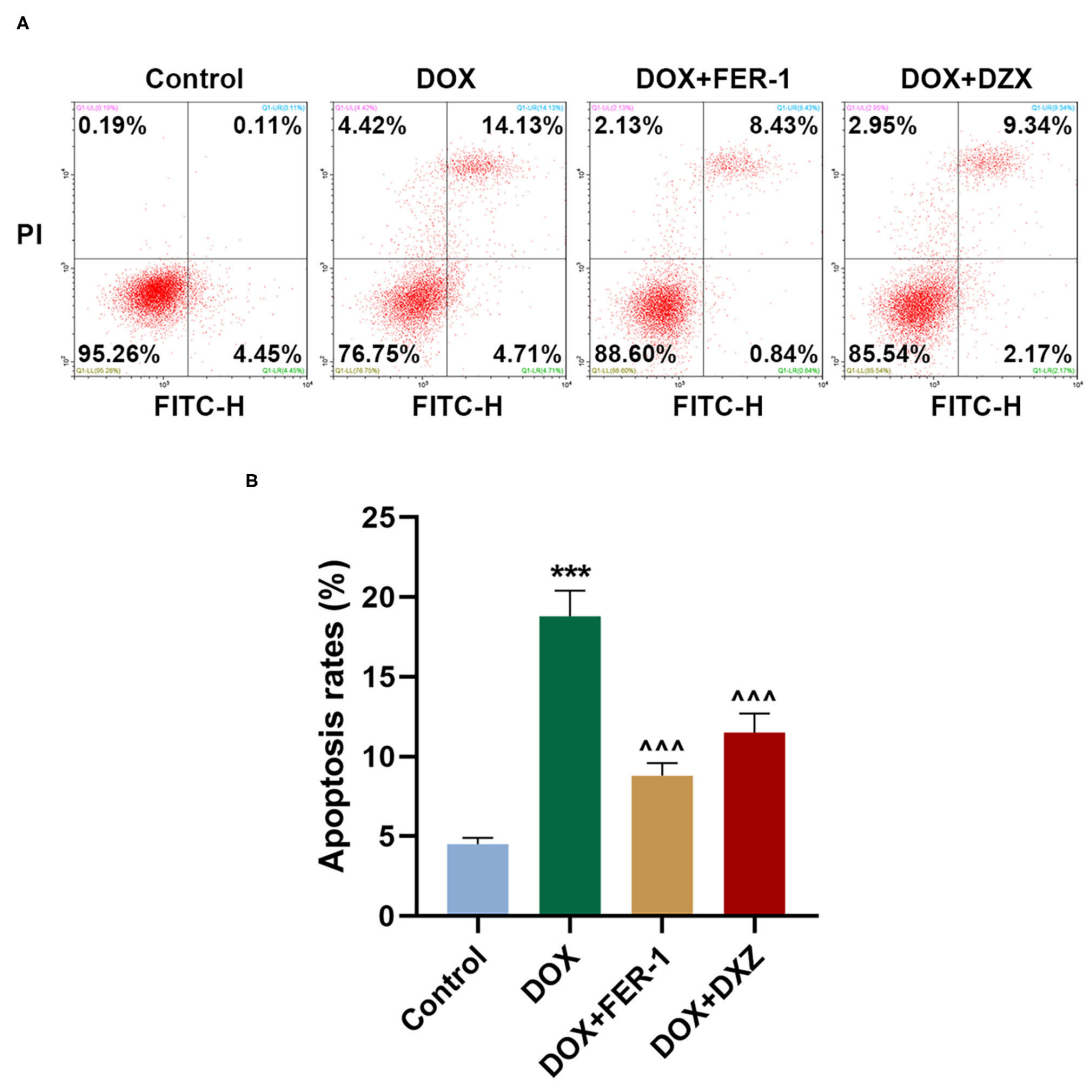
Effects of HMGB1 and DXZ on DOX-induced apoptosis of H9c2 rat cardiomyocytes. **(A,B)** The apoptosis rate of H9c2 cells after drug treatment was detected by flow cytometry. Left upper quadrant represents dead cells, left lower quadrant represents live cells, right upper quadrant represents late apoptotic cells, and right lower quadrant represents early apoptotic cells. The apoptotic populations of cells were calculated according to right upper and lower quadrant. ***P < 0.001 vs. control. ^∧∧∧^*P* < 0.001 vs. DOX. All experiments were repeated three times.

**Figure 9:**
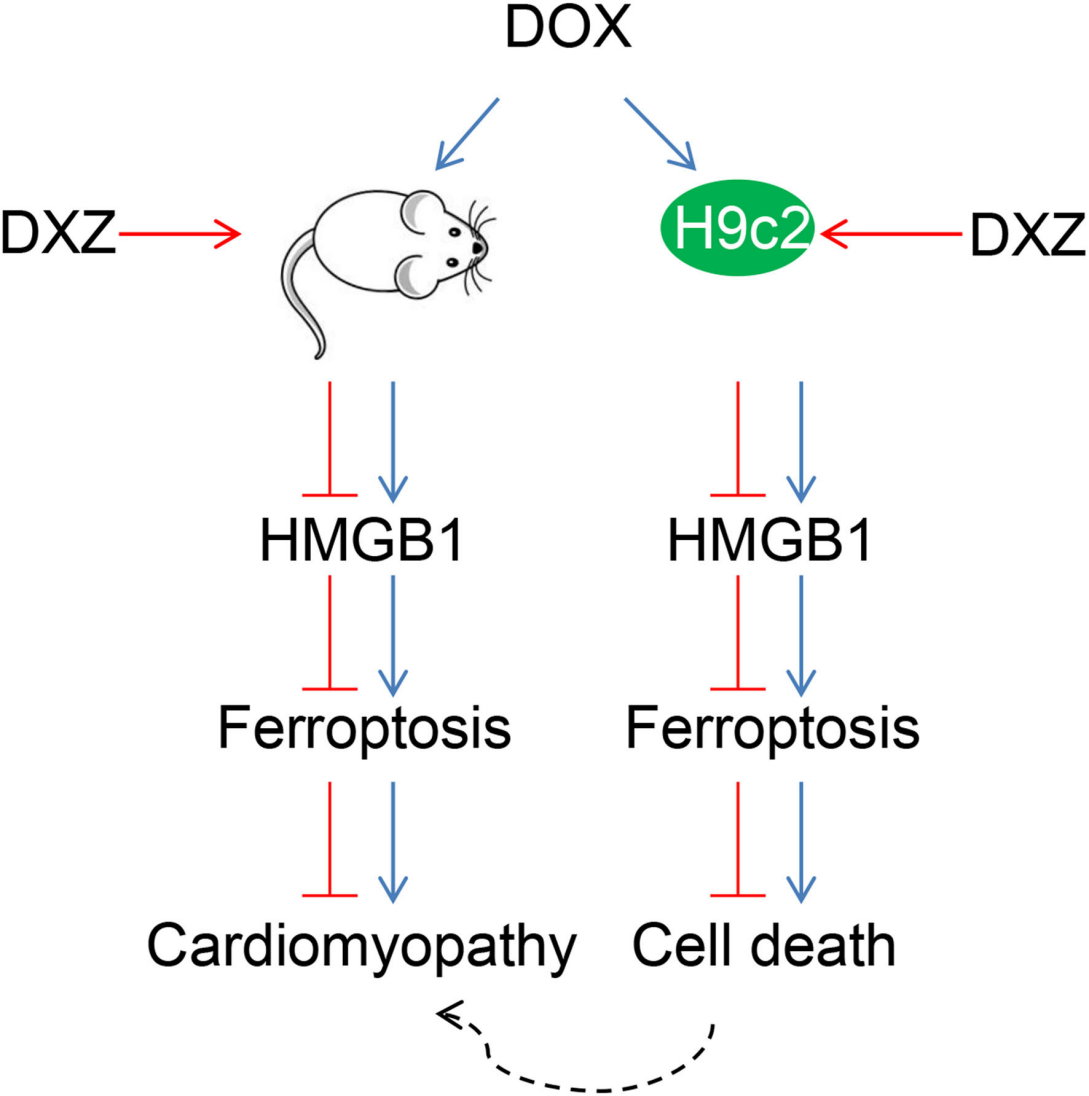
Protective effects of DXZ on DOX-induced rat cardiomyopathy through regulating HMGB1. A brief description of the mechanisms involved in this study that DXZ targeting HMGB1 inhibited ferroptosis of H9c2 cells and alleviated DOX-induced heart failure in rats.

Rat cardiomyocyte H9c2 was used to explore the effects of HMGB1 and DXZ on the ferroptosis and cardiotoxicity *in vitro*. H9c2 cells were incubated with DOX for 12 h at various doses [0 (control), 0.5, 1, 2, and 4 μM], and the results from the MTT assay indicate that DOX at the doses of 2 and 4 μM strongly suppressed viability of H9c2 cells (Figure 6A, *P* < 0.001). The MDA content of H9c2 cells was also induced by DOX at 2 and 4 μM (Figure 6B, *P* < 0.001). Intracellular Fe^2+^ was induced by DOX at 1, 2 and 4 μM (Figure 6C, *P* < 0.001). Next, the cells were exposed to DOX (2 μM) and incubated with FER-1 (10 μM), zVAD (20 μM), or DXZ (100 μM), and it could be found that the viability of H9c2 cells was suppressed by DOX, and the level of MDA and LDH release in H9c2 cells was increased by DOX. Moreover, the incubation with FER-1 or DXZ could rescue the reduction of viability and the increase of MDA and LDH release in H9c2 cells caused by DOX (Figures 6D–F, *P* < 0.001). Moreover, it could be found that zVAD, which is an apoptosis inhibitor, had no protective effect on the viability of DOX-treated H9c2 cells (Figure 6D). Incubation with FER-1 or DXZ could rescue the increase of Fe^2+^ in H9c2 cells caused by DOX (Figure 7A, *P* < 0.01). It is reported that the inhibition of glutathione peroxidase 4 (GPX4) induces ferroptosis (32); thus, the protein expressions of PTGS2, GPX4, and FTH1 were detected by Western blot analysis in H9c2 cells, the results of which demonstrate that DOX induced the increased expression of PTGS2 but decreased those of GPX4 and FTH1 (Figures 7B,C, *P* < 0.001). Also, FER-1 and DXZ were found to abolish the effects of DOX on the expressions of ferroptosis-related markers in H9c2 cells (Figures 7B,C, *P* < 0.001). Next, the Western blot results found that DOX induced the increased expression of HMGB1 protein, and DXZ was found to abolish the effects of DOX on the expressions of HMGB1 although FER-1 has no significant effect on it (Figures 7D,E, *P* < 0.001). Furthermore, we found that DOX induced the increased apoptosis of H9c2 cells, and FER-1 and DXZ were found to abolish the effects of DOX on cell apoptosis (Figures 8A,B, *P* < 0.001). The mechanistic figure with a brief description is shown in Figure 9: the results of *in vivo* and *in vitro* experiments demonstrate that DXZ targeting HMGB1 inhibited ferroptosis of H9c2 cells and alleviates DOX-induced heart failure in rats.

## Discussion

Chemotherapy using DOX can induce cardiomyopathy (33), and oxidative/nitrosative stress is one of the mechanisms that is related to the development of DOX-induced cardiotoxicity (34). However, it is demonstrated in a recent report that ferroptosis is also possibly the main cause of DOX-induced death (14). It was found in the current research that DXZ had a protective effect on the ferroptosis and cardiomyopathy in rats, which is consistent with our hypothesis that ferroptosis might be involved in the function of DXZ in DOX-induced cardiomyopathy of rats. Furthermore, it is also revealed in the results that HMGB1 might be a downstream regulator of DXZ and ferroptosis.

FER-1 is an arylalkylamine and has been used as an inhibitor for ferroptosis to inhibit cell death in multiple cell models, such as Huntington's disease, periventricular leukomalacia, and kidney dysfunction (35). FER-1 inhibits lipid peroxidation rather than mitochondrial ROS or lysosomal membrane permeability (35). According to the results in the current study, FER-1 could increase the survival time of DOX-treated rats. Moreover, other cell death–associated inhibitors, including NEC (a necrosis inhibitor), 3-MA (an autophagy inhibitor), and Emricasan (a pan-caspase inhibitor), had no positive effects on survival time of DOX-treated rats, suggesting that ferroptosis might be the main factor responsible for DOX-induced death in rats. Further experiments indicate that PTGS2, a molecular marker of ferroptosis, was increased by DOX in the heart, liver, and kidney of the rats, further confirming that ferroptosis was induced in DOX-treated rats. We also observed that PTGS2 was upregulated in the heart mostly by DOX, the results of which suggest that the heart might be the main affected organ in DOX-treated rats. Additionally, there was no change of PTGS2 expression in other organs, including in lung, brain, spleen, and pancreas, which is consistent with previous research (36).

It is further evidenced that DXZ had similar effects to FER-1 on the DOX-induced rats as the treatments with both FER-1 and DXZ can reduce the cardiomyopathy induced by DOX; block the increase in the expression of PTGS2 and the markers of cardiac hypertrophy; and reduce the expressions of ANP, BNP, and MYH7 in the heart tissue induced by DOX. Cardiac function was also improved by treatment of FER-1 or DXZ in DOX-induced rats. Those results, thus, suggest that ferroptosis might be involved in the protective effects of DXZ on DOX-induced cardiomyopathy.

Next, the molecular mechanism of DXZ in regulating ferroptosis and cardiomyopathy was explored. HMGB1 encodes a protein that belongs to the high mobility group-box superfamily and plays an important role in several cellular processes, including inflammation, cell differentiation, and tumor cell migration (37–39). An elevated HMGB1 level is predictive of myocardial injury and is also associated with poor clinical outcomes (40). Moreover, it has been previously discovered that the suppression of HMGB1 has a protective effect on hyperglycemia-induced apoptosis and mitochondrial dysfunction in cardiomyocytes (41, 42). It was also revealed recently that the balance between senescence and apoptosis in response to DOX-induced cytotoxicity can be regulated by HMGB1 (43). Furthermore, it is proposed that the protective effect of HMGB1 on DOX-induced cardiotoxicity is associated with the regulation of autophagy (44). Moreover, HMGB1 is also closely related to ferroptosis, and to be specific, inhibition of HMGB1 significantly reduces the degree of ferroptosis during acute liver failure (21). However, at present, it is not clear whether the protective effects of DXZ on DOX-induced cardiomyopathy are related to the regulation of HMGB1. Thus, we speculated that HMGB1 might be involved in the protective effects of DXZ on DOX-induced cardiomyopathy. In this research, we found that HMGB1 was indeed involved in DOX-induced cardiotoxicity. Mechanically, it is considered that autophagy-mediated inhibition of histone deacetylase promotes the acetylation of HMGB1, thus resulting in the release of HMGB1 during ferroptosis (45). However, in accordance with our previous results, the autophagy inhibitor had no effect on the survival of DOX-treated rats. Thus, whether the autophagy inhibitor affects the expression of HMGB1 in DOX-treated rats should be further confirmed.

Heme homeostasis is involved in many redox activity–associated biological processes (46), and the degradation of heme and the release of free iron (non-heme iron) can be induced by DOX, ultimately causing ferroptosis and heart failure (14). Consistently, we found that DOX reduced the level of heme in heart tissues and serum of rats, and the level of non-heme iron was increased in indicated organs and serum of rats treated by DOX. Furthermore, the protective role of DXZ in DOX-induced ferroptosis and cardiotoxicity was also confirmed in experiments *in vitro*, where rat cardiomyocyte H9c2 was treated by DOX with FER-1, zVAD, or DXZ, the results of which were consistent with the results *in vivo*. In this study, we also found that DOX inhibited cell viability and promoted lipid peroxidation in a dose-dependent manner, the effects of which were obvious after the cardiomyocytes were treated with over 1 μM DOX. Meanwhile, DOX promoted the content of Fe^2+^ in cells in a dose-dependent manner. The slight difference in the different experimental results of the lower concentration of DOX may be due to the different experimental analytical methods, but the overall trend is consistent, showing the myocardial cytotoxicity of DOX. Based on the experiments *in vivo*, we found that DOX treatment induced cardiac hypertrophy, and such effect was diminished by the treatment using DXZ or FER-1 on the heart. As pathological cardiac hypertrophy is relevant to the apoptosis in the cardiomyocyte (47), therefore, we further detected the apoptosis rate of cells in experiments *in vitro*. In addition, FER-1 and DXZ can decrease the content of Fe^2+^ and the apoptosis in cells after DOX treatment, which further verifies the protective effects of DXZ on myocardial cells. In accordance with the results in our experiment, the regulation of intracellular Fe^2+^, cell apoptosis, and ferroptosis-related proteins under the effect of FER-1 and DXZ were similar, which further suggests that the effect of DXZ in DOX-induced cardiomyopathy is mainly dependent on the regulation of ferroptosis. Additionally, it is noted that the protein level of FTH1, an iron-related gene, was decreased by DOX but was increased by DXZ; however, the mRNA level of FTH1 in DOX-induced rats was increased, which was similar to a previous study (48). Based on the results of qRT-PCR analysis, the trend on the mRNA expression of TFRC was opposite that of FTH1 and BNP, from which we speculated that the difference was probably caused by the deviation in Western blotting and qRT-PCR analysis or a more complicated gene regulation relationship in ferroptosis, which, if so, should be further determined. However, there are some limitations in this study: Ferroptosis is closely related to mitochondria, and we will supplement this part in a future study. We will supplement HMGB1 silencing *in vitro* experiments to explore the role of HMGB1 in a more comprehensive way.

In conclusion, the current study uncovers a mechanism through which DXZ protects against the DOX-induced cardiotoxicity through regulating ferroptosis *via* HMGB1. It is suggested in our findings that DOX in combination with DXZ could be a feasible therapeutic approach for managing DOX-induced cardiac injury without compromising the anticancer properties of the drug. Moreover, future studies are required to test the potential clinical implications of DXZ.

## Data Availability Statement

The original contributions presented in the study are included in the article/supplementary material, further inquiries can be directed to the corresponding author.

## Ethics Statement

The animal study was reviewed and approved by the Animal Research Ethics Committee of the Second Affiliated Hospital, Nanjing Medical University (No. H2016051930W).

## Author Contributions

HZ and ZW: substantial contributions to conception and design, drafting the article, or critically revising it for important intellectual content. ZL, KD, and XL: data acquisition, data analysis, and interpretation. All authors: final approval of the version to be published, agreement to be accountable for all aspects of the work in ensuring that questions related to the accuracy, or integrity of the work are appropriately investigated and resolved.

## Conflict of Interest

The authors declare that the research was conducted in the absence of any commercial or financial relationships that could be construed as a potential conflict of interest.
